# Wie steht es um die Mundgesundheit in Deutschland?

**DOI:** 10.1007/s00103-021-03357-2

**Published:** 2021-06-29

**Authors:** Ingrid Schubert, Stefan Listl

**Affiliations:** 1 PMV forschungsgruppe an der Klinik und Poliklinik für Psychiatrie, Psychosomatik und Psychotherapie des Kindes- und Jugendalters, Medizinische Fakultät und Uniklinik Köln, Universität zu Köln, Köln, Deutschland; 2 Department of Dentistry, Chair for Quality and Safety of Oral Health Care, Radboud Universitair Medisch Centrum, Nijmegen, Niederlande; 3 Sektion für Translationale Gesundheitsökonomie, Poliklinik für Zahnerhaltungskunde, Universitätsklinikum Heidelberg, Heidelberg, Deutschland

Als wir im November 2019 dem Herausgeberbeirat den Vorschlag unterbreiteten, ein Schwerpunktheft zum Thema Mundgesundheit zu koordinieren, erhielten wir große Zustimmung und zahlreiche Themenvorschläge. Daraus sind jetzt zwei Ausgaben (Juli und August 2021) entstanden. Die Relevanz der Thematik zeigt sich nicht nur in der Tatsache, dass Mundgesundheit mit Lebensqualität korreliert, und in dem hohen Anteil der Leistungsausgaben der gesetzlichen Krankenversicherung, die auf die vertragszahnärztliche Versorgung entfallen. Das Thema ist auch deshalb von hoher Bedeutung für Public Health, da wesentliche orale Erkrankungen der Verhaltensprävention und – politischer Wille vorausgesetzt – auch der Verhältnisprävention zugänglich sind.

Weltweit erfährt die Mundgesundheit derzeit zunehmende Aufmerksamkeit. Der WHO-Exekutivrat verabschiedete Anfang 2021 eine bahnbrechende Resolution zur Mundgesundheit [1]. Fast gleichzeitig veröffentlichte der Weltzahnärzteverband das Strategiepapier „FDI Vision 2030“ [2]. Sowohl die WHO-Resolution als auch die „FDI Vision 2030“ betonen die Notwendigkeit, die Mundgesundheit als essenziellen Bestandteil der universellen Gesundheitsversorgung zu stärken. Beide Berichte verweisen übrigens auch umfangreich auf Ergebnisse zahnmedizinischer Versorgungsforschung aus Deutschland (z. B. zu den ökonomischen Auswirkungen von Zahnerkrankungen).

Im internationalen Vergleich haben wir in Deutschland eine gute zahnmedizinische Versorgung. Dennoch besteht Optimierungsbedarf. Vergleichbar der Diskussion in anderen medizinischen Fachbereichen geht es auch in der Zahnmedizin unter anderem um die folgenden Aspekte:

- Verbesserung der Mundgesundheitskompetenz der Bevölkerung (Health Literacy),
- Qualitätsförderung auf Basis sinnvoller Qualitätsindikatoren sowie Reflexion bzgl. der Vermeidung von Unter‑, Über- und Fehlversorgung,
- Strukturen und Prozesse in der Versorgung, z. B. für die Versorgung multimorbider und sozial benachteiligter Personen, von Menschen in Pflegesettings und von Menschen mit Behinderung,
- zum Teil unklare Evidenzbasierung der Versorgung: Nicht immer ist der Nutzen zahnmedizinischer Maßnahmen ausreichend durch wissenschaftliche Evidenz belegt.
- Die Weiterentwicklung einer bedarfsgerechten und finanzierbaren Versorgung, die für alle zugänglich ist. Dies setzt Kenntnisse über Versorgungsbedarfe und Barrieren im Zugang zur Versorgung aus der Perspektive von Nutzerinnen und Nutzer voraus.
- Eine bedarfsgerechte Versorgungsplanung erfordert neben geeigneten epidemiologischen Daten auch die evidenzbasierte Implementierung neuer Versorgungsformen sowie der dafür erforderlichen Strukturen und Prozesse in der zahnmedizinischen Versorgung. Hier steht die Versorgungsforschung in der Zahnmedizin noch am Anfang.
- Entwicklung von Konzepten und Routinen interprofessioneller Versorgung als Antwort auf die Erkenntnis, dass bei einigen chronisch systemischen Erkrankungen ein Zusammenhang mit der Mundgesundheit besteht,
- Umgang mit Interessenskonflikten (z. B. Zuckerindustrie, Werbung, Dentalindustrie),
- Herausforderungen im Zusammenhang mit der Digitalisierung (Telematikinfrastruktur, Entscheidungsunterstützungssysteme, mobile Applikationen etc.) und nicht zuletzt
- fortwährende Weiterentwicklung von Aus‑, Fort- und Weiterbildung sowie nachhaltiger Ausbau geeigneter Strukturen für zahnmedizinische Versorgungsforschung an den Universitäten.

Einige der hier genannten Punkte werden in den Beiträgen dieses Heftes oder im Folgeheft aufgegriffen.

Die ersten sechs Beiträge fokussieren auf die Mundgesundheit in ausgewählten Populationen. *Schmoeckel et al.* präsentieren epidemiologische Daten zur Mundgesundheit bei Kindern verschiedener Altersgruppen. Zwar ist bei den 12-Jährigen eine deutliche Verbesserung hinsichtlich der Karieshäufigkeit zu beobachten, Sorge bereitet jedoch die Situation im Milchgebiss. Hier sieht die Autorengruppe noch deutlichen Verbesserungsbedarf und hält einen stringenten evidenzbasierten Handlungsplan „Prävention im Milchgebiss“ in Deutschland für sinnvoll.

*Kocher et al.* nehmen die Erwachsenen in den Blick und berichten auf der Basis großer bevölkerungsbezogener Studien, wie sich die Prävalenzen der Karies, der Parodontitis und des Zahnverlustes von 1997 bis 2014 in Deutschland verändert haben. Sie konstatieren eine deutliche Verbesserung der oralen Gesundheit durch eine verbesserte Mundhygiene, erwarten jedoch aufgrund der Altersstruktur der Bevölkerung eine Zunahme parodontaler Erkrankungen.

Eine deutlich eingeschränktere Datenbasis liegt zur Mundgesundheit bei Menschen mit Behinderung vor. *Schulte und Schmidt* zeigen literaturgestützt die bestehende Benachteiligung. Sie sehen die Notwendigkeit, bereits in der zahnmedizinischen Ausbildung auf die Versorgung von Patienten mit Behinderung vorzubereiten. Diesen Aspekt greifen auch *Nitschke und Hahnel* für ältere Menschen mit Pflegebedarf auf, die ein höheres Risiko für eine schlechte Mundgesundheit im Vergleich zur Durchschnittsbevölkerung aufweisen. Aus ihrer Sicht sollte ein aufsuchendes Versorgungskonzept für Menschen in Pflege in den unterschiedlichsten Settings sichergestellt werden.

Eine Übersicht zur gesundheitlichen Lage der Bevölkerung und ihrer Bewertung geben die Gesundheitsberichte auf der Ebene des Bundes, der Länder und Kommunen. Mundgesundheit wird in diesen Berichten teilweise adressiert, doch fehlen, wie *Lüders et al.* zeigen, oftmals Daten, insbesondere für vulnerable Gruppen auf kleinräumiger Ebene sowie entsprechende Indikatorensätze für ein umfassendes Monitoring.

Epidemiologische Daten zur Mundgesundheit bilden auch die Basis für die Formulierung von Gesundheitszielen sowie für die Überprüfung der Zielerreichung. *Ziller et al.* stellen Mundgesundheitsziele 2030 mit Handlungsempfehlungen aus Sicht der Zahnärzteschaft vor. Ob die Zielerreichung gelingen wird, ist auch von den gesundheitspolitischen Rahmenbedingungen der Präventions- und Gesundheitspolitik abhängig.

Vier weitere Beiträge befassen sich mit ausgewählten Maßnahmen in der Zahnmedizin. *U. Schiffner* erläutert den Nutzen von Fluoriden in der Kariesprävention als wichtigen Bestandteil eines oralpräventiven Gesamtkonzeptes, zu dem auch eine ausgewogene und möglichst zuckerarme Ernährung gehört. Den letztgenannten Aspekt greifen *Heilmann und Ziller* in ihrem Beitrag auf und benennen eine Reihe von Faktoren, die den Zuckerkonsum beeinflussen. Sie betonen, dass es zwar weiterhin der Gesundheitsaufklärung und Maßnahmen zur individuellen Verhaltensänderung bedarf, diese allein in der Regel jedoch wenig Wirkung zeigen und dringend durch wirtschafts- und gesundheitspolitische Maßnahmen flankiert werden müssen.

Auch wenn der Einsatz von Amalgam in den letzten 30 Jahren deutlich zurückgegangen ist, sind Vor- und Nachteile zu diskutieren und Versorgungsalternativen ebenfalls kritisch zu beleuchten. Diese Diskussion zeichnet der Beitrag von *Frankenberger et al.* fundiert nach. Die Autorengruppe plädiert, eine Entscheidung für oder gegen die Verwendung von Amalgam patientenindividuell vorzunehmen. Während die Diskussion um Amalgam auch Nichtzahnmedizinern bekannt ist, dürfte die Frage zur Freisetzung von Aluminium aus Glitzerpartikeln bei herausnehmbaren kieferorthopädischen Apparaturen bislang eher fachintern diskutiert worden sein. *Wepner et al.* geben einen kurzen Einblick in die Thematik.

In diesem ersten Heft spannen wir den Bogen von der Epidemiologie über Versorgung hin zur zahnmedizinischen Versorgungsforschung. *Hüttig und Schwendicke* beschreiben Stand und zukünftige Entwicklung für eine erfolgreiche Versorgungsforschung in der Zahn‑, Mund- und Kieferheilkunde. Ausgehend von der vom WHO-Exekutivrat verabschiedeten Resolution zur Mundgesundheit (s. oben) benennen sie Versorgungsbedarf und wichtige Themenfelder zukünftiger Versorgungsforschung in der Zahnmedizin wie Versorgungsgerechtigkeit und Nachhaltigkeit.

*Benzian und Listl* thematisieren Herausforderungen und Chancen für die Mundgesundheit im globalen Kontext. Mit fast 3,5 Mrd. Erkrankungsfällen sind so viele Menschen von oralen Erkrankungen betroffen wie von keiner anderen Krankheitsgruppe. Dennoch wird der Mundgesundheit im gesundheitspolitischen Diskurs bislang nur wenig Priorität eingeräumt. Ausgehend von einer kürzlich veröffentlichten *Lancet*-Artikelserie zur globalen Mundgesundheit stellt der Beitrag die im internationalen Diskurs wichtigen Themen sowie Verbesserungsansätze dar.

Der Reigen an Beiträgen schließt mit einem Plädoyer von *H. Strippel* für eine konsequente Umsetzung einer Public-Health-Perspektive zur Förderung der Mundgesundheit. Es bedarf mundgesundheitsförderlicher Veränderungen in sozialen und ökonomischen Lebenswelten, worauf in einigen anderen Beiträgen ebenfalls hingewiesen wurde. Ob dies durch eine neu zu etablierende Institution oder durch gemeinschaftliches Handeln der vorhandenen Akteure erreicht wird, bleibt zu diskutieren.

Wir danken allen Autorinnen und Autoren für ihre Beiträge wie auch den Gutachterinnen und Gutachtern, die wertvolle Anregungen gegeben haben, und wünschen eine anregende Lektüre.
